# Supplementary material for: Comparison between Some Phenotypic and Genotypic Methods for Assessment of Antimicrobial Resistance Trend of Bovine Mastitis Staphylococcus aureus Isolates from Bulgaria
Source: Vet Sci. 2022 Jul 31;9(8):401. doi: 10.3390/vetsci9080401 (PMC9416698; doi:10.3390/vetsci9080401)
Supplement: Supplementary file 1 [file vetsci-09-00401-s001.zip › vetsci-1812514-supplementary.pdf]

Table S1. *Nuc* PCR temperature conditions

Steps	Temperature	Exposition
Initial denaturation	95°C	5 min
Denaturation	95°C	30 sec
Annealing	55°C	30 sec
Elongation	72°C	30 sec
Final elongation	72°C	7 min
Cycles	30	-

Table S2. Classes of antimicrobial drugs included in the mastitis pathogens testing plate (Trek Diagnostic Systems Ltd, East Grinstead, UK), their concentrations and susceptible MIC breakpoints according to CLSI [27].

Antimicrobials	Concentrations in the plate μg/mL	Susceptible MIC breakpoint μg/mL
**Beta-lactams/penicillins**		
Penicillin	0.12 - 8	≤ 0.12
Ampicillin	0.12 - 8	≤ 0.25
Oxacillin	2 - 4	≤ 2
**Beta-lactams/other class combinations**		
Penicillin/novobiocin	1/2 – 8/16	≤ 1/2
**Beta-lactams/cephalosporins**		
Cephalothin – 1st generation	2 - 16	≤ 8
Ceftiofur – 3rd generation	0.5 - 4	≤ 2
**Macrolides**		
Erythromycin	0.25 - 4	≤ 0.5
**Lincosamides**		
Pirlimycin	0.5 - 4	≤ 2
**Tetracyclines**		
Tetracycline	1 - 8	≤ 4
**Sulfonamides**		
Sulfadimethoxine	32 - 256	≤ 256

Table S3. Sequence of primers, targeted genes, expected size of PCR products and their source.

Gene	Sequence (5′–3′)	Size, bp	Source
blaZ	1-AAG AGA TTT GCC TAT GCT TC<br>2-GCT TGA CCA CTT TTA TCA GC	517	[19]
***erm*B**	1-ACG ACG AAA CTG GCT AA<br>2-TGG TAT GGC GGG TAA	409	
***erm*C**	1-CTT GTT GAT CAC GAT AAT TTC C<br>2-ATC TTT TAG CAA ACC CGT ATT C	190	
***tet*K**	1-TCG ATA GGA ACA GCA GTA<br>2-CAG CAG ATC CTA CTC CTT	169	
***tet*M**	1-CCG CAC CCT CTA CTA CAA<br>2-CAT TCC ACT TCC CAA CG	351	
***mec*A**	1-GTA GAA ATG ACT GAA CGT CCG ATA A<br>2-CCA ATT CCA CAT TGT TTC GGT CTA A	310	[28]

Table S4. Primary identification of bovine mastitis *Staphylococcus* spp. isolates.

Tests								Isolates n/%
Catalase	Oxidase	Haemolysis			Pigment	Mannitol	Coagulase	
		Double	Beta	Gamma				
+	-	+			yellowish	+	+	62/67.4
+	-	+			yellowish	+/-	+	1/1.1
+	-		+		yellowish	+	+	6/6.5
+	-		+		greyish	+	+	12/13.0
+	-		+		greyish	+	-	5/5.4
+	-		+		greyish	+/-	-	3/3.3
+	-			+	greyish	+/-	+	3/3.3

+/- = weakly positive reaction

Table S5. Distribution of isolates resistant in the Bauer-Kirby disk diffusion test by regions, districts and farms

Region	No farm	N Districts	N isolates	N resistant isolates
**North central**	1	Veliko Tarnovo	8	2
	2	Silistra	2	1
**Northeast**	3	Targovishte	4	3
**South central**	4	Plovdiv	13	7
	5	Kardzhali	5	0
	6	Pazardzhik	2	0
**Southeast**	7	Stara Zagora	5	3
	8	Stara Zagora	3	2
	9	Stara Zagora	4	0
	10	Stara Zagora	13	7
	11	Stara Zagora	12	5
	12	Sliven	3	2
	13	Sliven	14	0
	14	Burgas	4	0
**Total**	**14**	**9**	**92**	**32**

Table S6. Resistance patterns of bovine mastitis *S. aureus* isolates resistant in the Bauer-Kirby disk diffusion test by regions, districts and farms

Antimicrobial resistance patterns	Region	District	No Farm	N isolates
**S**	Southeastern	Stara Zagora	10	1
			11	2
**P (A)**	North central	Veliko Tarnovo	1	2
		Silistra	2	1
	Northeastern	Targovishte	3	1
	South central	Plovdiv	4	1
	Southeastern	Sliven	12	2
		Stara Zagora	7	3
			8	2
			10	5
			11	1
**P (A)-E**	South central	Plovdiv	4	1
	Southeastern	Stara Zagora	11	2
**P (A)-T**	Northeastern	Targovishte	3	1
**P (A)-T-E**	South central	Plovdiv	4	1
	Southeastern	Stara Zagora	10	1
**P (A)-S-E**	South central	Plovdiv	4	2
**P (A)-S-T-E**	South central	Plovdiv	4	2
**P (A)-EFT-S-G-T**	Northeastern	Targovishte	3	1
**Total**				**32**

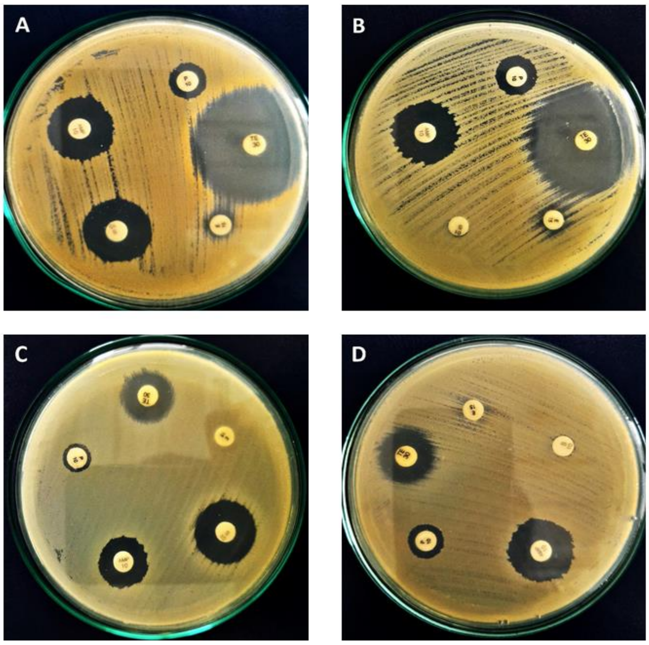

Figure S1. Some resistance patterns depicted by the disk diffusion test. A – resistance to penicillin, ampicillin and erythromycin; B – resistance to penicillin, ampicillin, streptomycin and erythromycin; C – resistance to penicillin, ampicillin, tetracycline and erythromycin; D – resistance to penicillin, ampicillin, streptomycin, tetracycline and erythromycin.

Table S7. Comparison between disk diffusion and MIC methods for testing resistance to penicillin.

	Minimum inhibitory concentration		Total
	N sensitive isolates	N resistant isolates	
Disk diffusion method N sensitive isolates	63	0	63
Disk diffusion test N resistant isolates	1	28	29
Total	64	28	92

Table S8. Comparison between disk diffusion and MIC methods for testing resistance to ampicillin.

	Minimum inhibitory concentration		Total
	N sensitive isolates	N resistant isolates	
Disk diffusion method N sensitive isolates	62	1	63
Disk diffusion test N resistant isolates	0	29	29
Total	62	30	92

Table S9. Comparison between disk diffusion and MIC methods for testing resistance to erythromycin.

	Minimum inhibitory concentration		Total
	N sensitive isolates	N resistant isolates	
Disk diffusion test N sensitive isolates	80	3	83
Disk diffusion test N resistant isolates	0	9	9
Total	80	12	92

Table S10. Comparison between disk diffusion and MIC methods for testing resistance to tetracycline.

	Minimum inhibitory concentration		Total
	N sensitive isolates	N resistant isolates	
Disk diffusion method N sensitive isolates	86	0	86
Disk diffusion test N resistant isolates	0	6	6
Total	86	6	92
